# Supplementary material for: TRPC6 Deficiency Attenuates Mitochondrial and Cardiac Dysfunction in Heart Failure with Preserved Ejection Fraction Induced by High-Fat Diet Plus L-NAME
Source: Int J Mol Sci. 2025 Sep 25;26(19):9383. doi: 10.3390/ijms26199383 (PMC12524895; doi:10.3390/ijms26199383)
Supplement: Supplementary file 1 [file ijms-26-09383-s001.zip › ijms-3624736-supplementary.pdf]

Supplemental Material

Methods:

Mitochondria isolation and purification: Heart and brain tissues were collected from WT mice. Crude mitochondria were isolated by the mitochondrial isolation kit (Novus, NBP2-29448) and then were magnetically labeled with Anti-TOM22 MicroBeads (130-127-693, Miltenyi Biotec), which bind to the translocase of the outer mitochondrial membrane 22 protein (TOM22). The sample is loaded onto an LS Column placed in a MidiMACS or QuadroMACS Separator. After washing, only magnetically labeled mitochondria are retained on the column. The column is removed from the separator, and functional mouse mitochondria are eluted from the column (130-096-946, Mitochondria Isolation Kit, Miltenyi Biotec).

Western blot: Purified mitochondrial pellet was lysed using a mitochondrial lysis buffer. After protein concentration was measured by BCA assay, a total 15 µg protein of whole cell lysate and mitochondria was separated in 7.5% to 12% SDS-polyacrylamide gels (Bio-rad, CA). After transfer to nitrocellulose membranes, blots were rinsed in PBS and blocked in Odyssey blocking buffer (LI-COR, NE) for 1 h at room temperature and then incubated with TRPC6 rabbit antibody (1:500, ACC-120, Alomone Labs), TRPC3 rabbit antibody (1:500, Cell signaling), VDAC rabbit antibody (1:1,000, Cell signaling), TrR rabbit antibody (1:1,000, Cell signaling), Syntaxin 6 rabbit antibody (1:1,000, Cell signaling), ERp72 rabbit antibody (1:1,000, Cell signaling), α-Tubulin rabbit antibody (1:3,000, Cell signaling) and β-actin rabbit antibody (1:3,000, Abcam) at 4 °C overnight. Membranes were probed with LI-COR fluorescent dye-labeled secondary antibodies (1:5,000) for 1 h at room temperature. Antibody labeling was visualized using the Odyssey Infrared Scanner (LI-COR, NE).

Figure S1

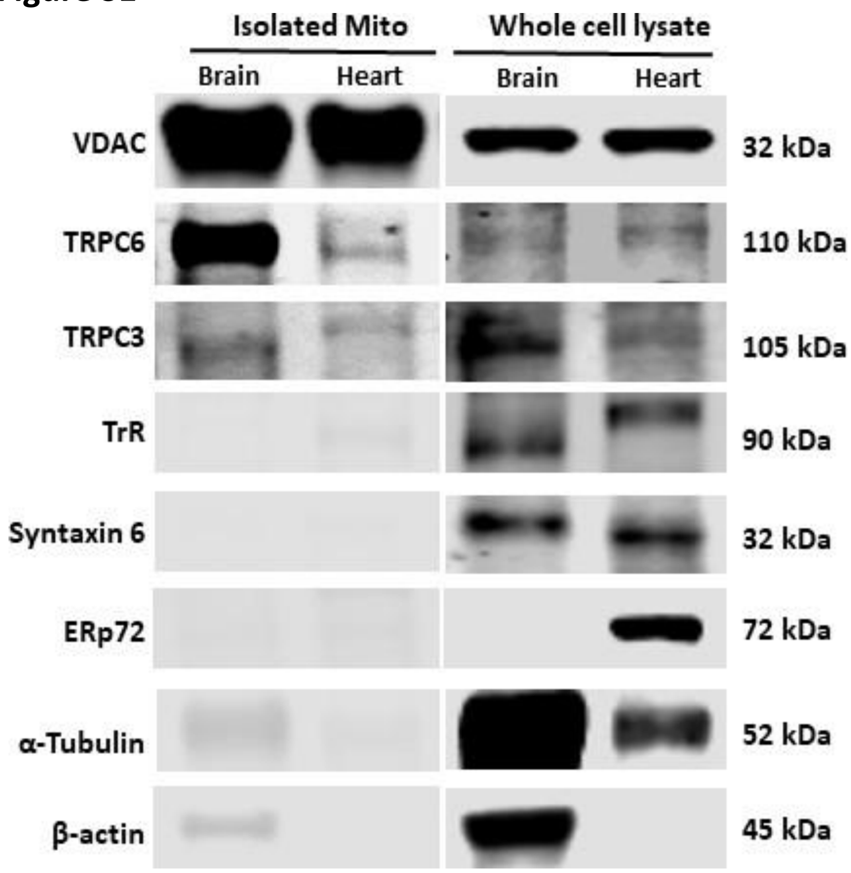

Figure S1. TRPC6 was detected by western blot in purified mitochondrial fractions and whole cell lysate from brain and cardiac tissue of WT mice. VDAC (voltage-dependent anion channel), TRPC3 as mitochondrial markers, TrR (Transferrin receptor) as a plasma membrane marker, Syntaxin 6 as a Golgi marker, ERp72 (ER stress protein 72) as an ER marker, and α-Tubulin and β-actin as plasma protein markers.

Figure S2

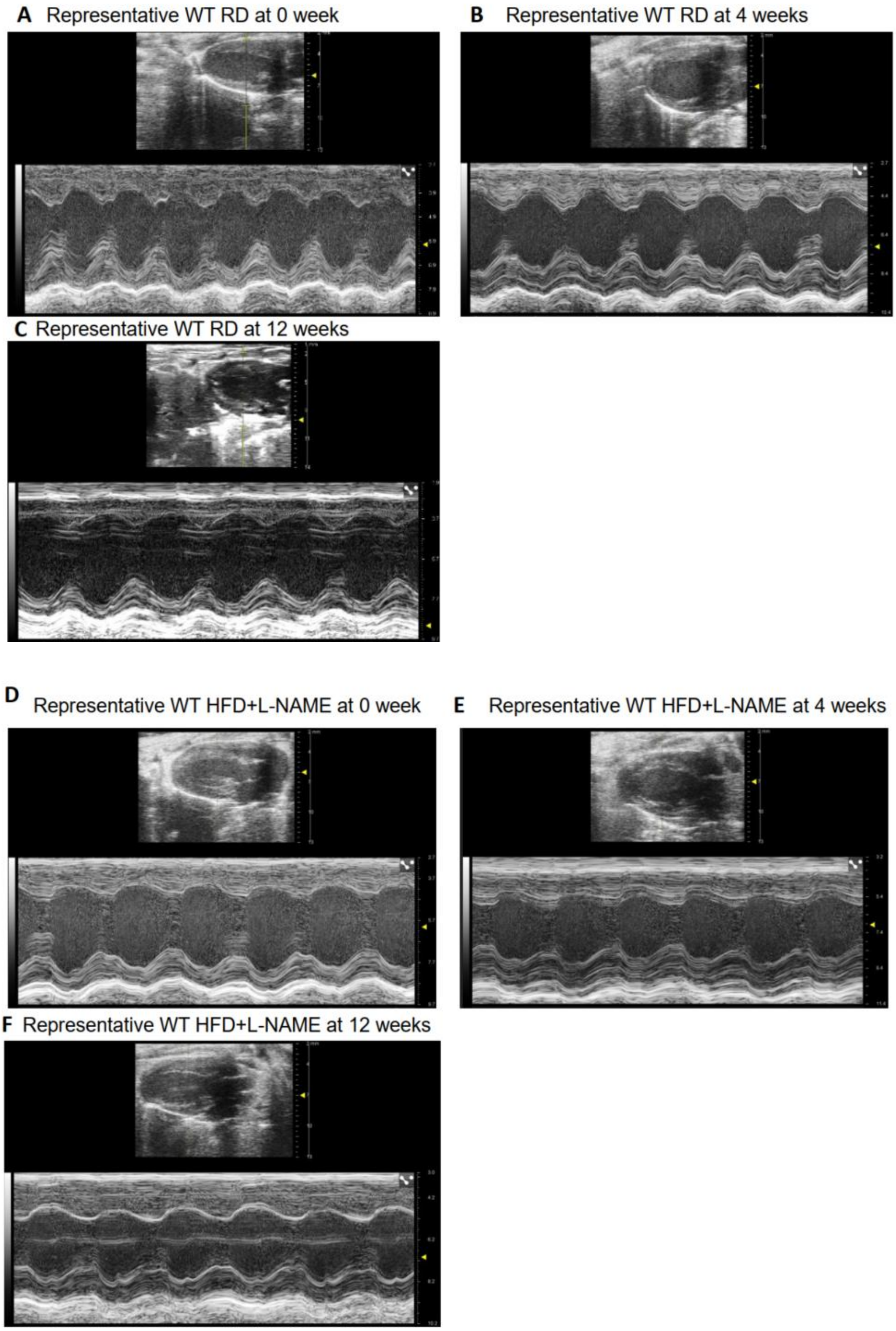
A Representative WT RD at 0 week
B Representative WT RD at 4 weeks
C Representative WT RD at 12 weeks
D Representative WT HFD+L-NAME at 0 week
E Representative WT HFD+L-NAME at 4 weeks
F Representative WT HFD+L-NAME at 12 weeks

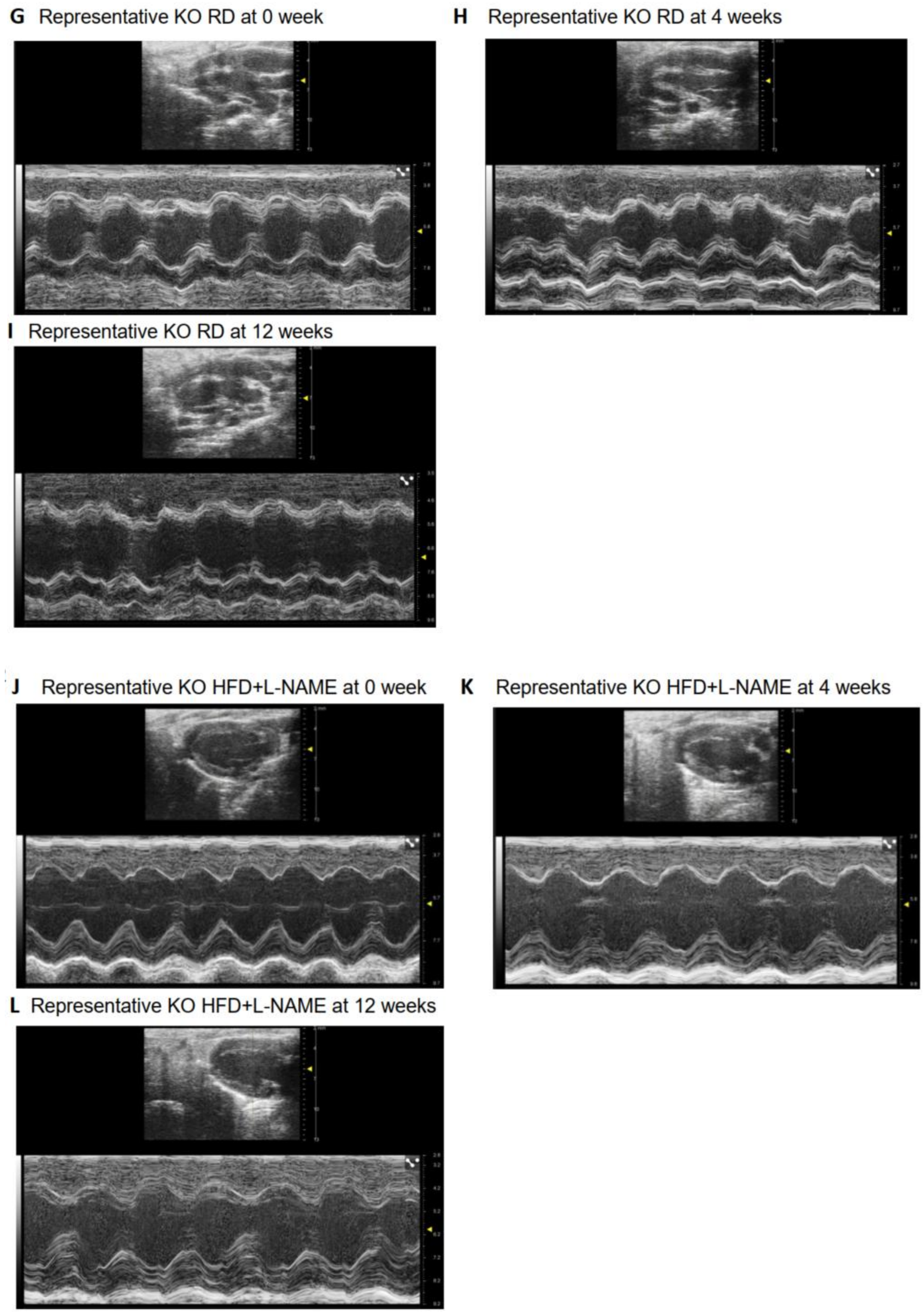

Figure S2. Representative echocardiographic B-mode (top) and M-mode (bottom) images of WT and TRPC6 KO mice over time. (A–C) WT mice with RD at 0, 4, and 12 weeks. (D–F) WT mice with HFD+L-NAME at 0, 4, and 12 weeks. (G–I) TRPC6 KO mice with RD at 0, 4, and 12 weeks. (J–L) TRPC6 KO mice with HFD+L-NAME at 0, 4, and 12 weeks.

Figure S3

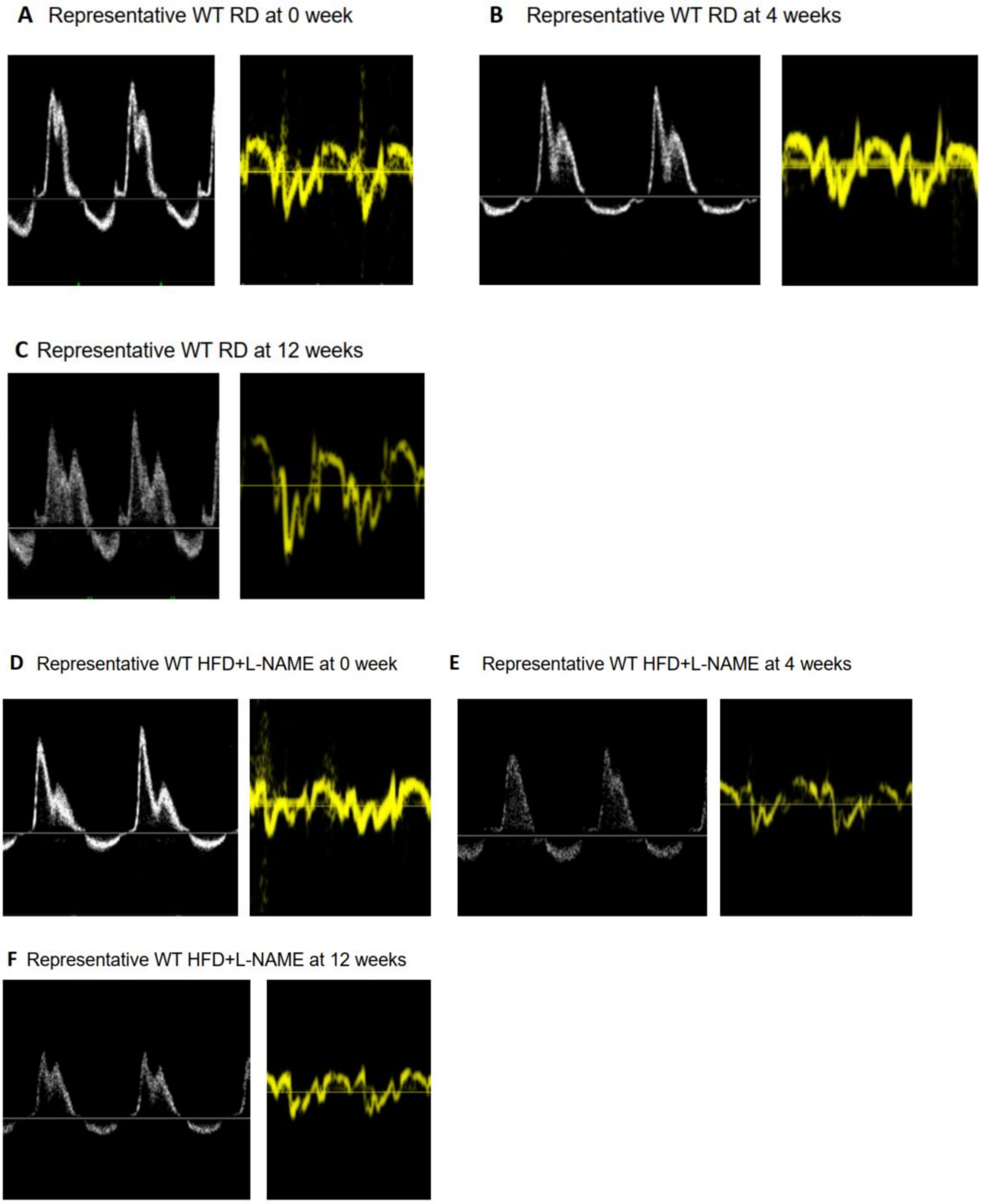
A Representative WT RD at 0 week
B Representative WT RD at 4 weeks
C Representative WT RD at 12 weeks
D Representative WT HFD+L-NAME at 0 week
E Representative WT HFD+L-NAME at 4 weeks
F Representative WT HFD+L-NAME at 12 weeks

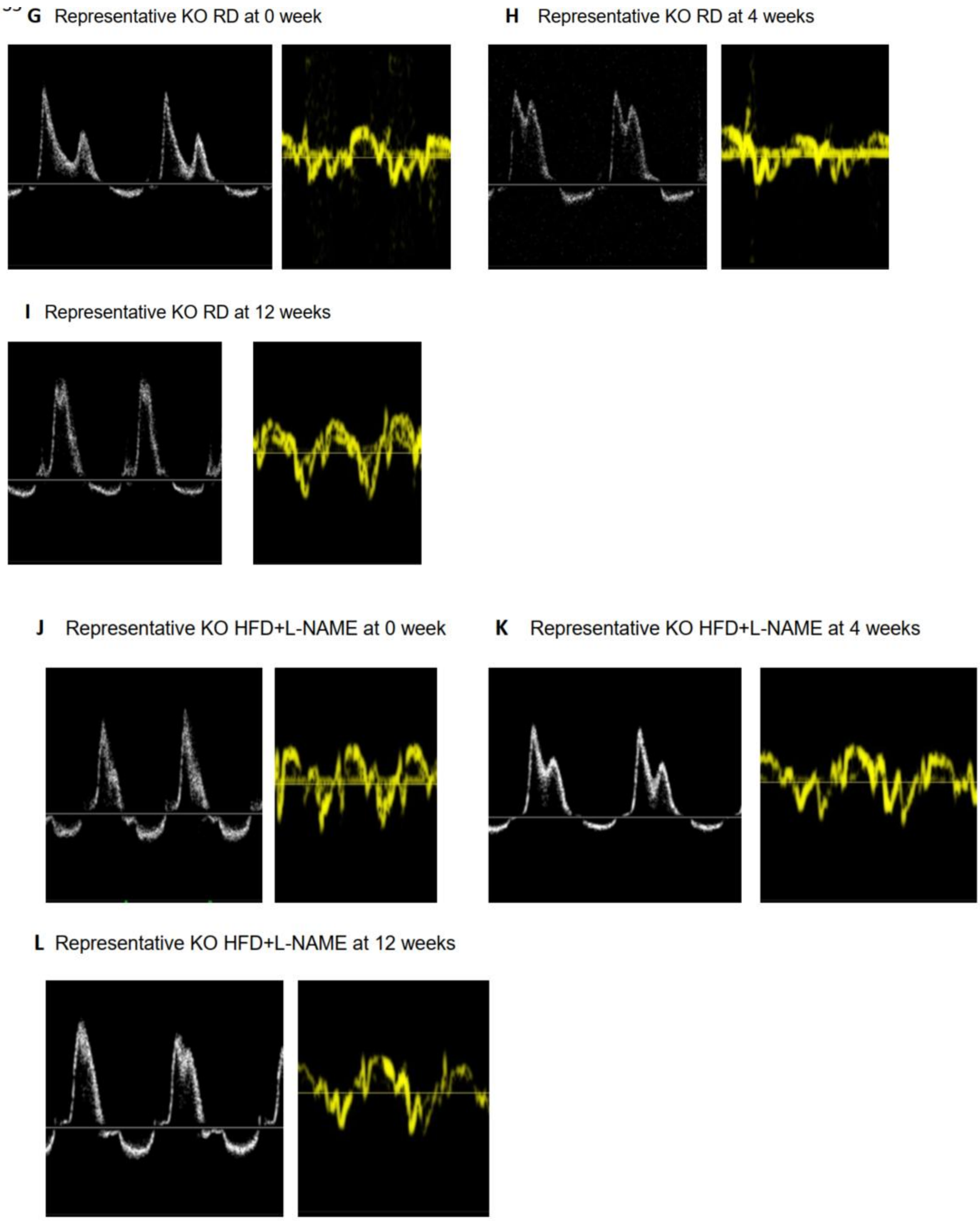

Figure S3. Representative Doppler echocardiographic images in different groups of WT and TRPC6 KO mice over time. Pulsed-wave Doppler (left) and tissue Doppler (right) recordings, demonstrating temporal changes in transmitral inflow and myocardial velocities. (A–C) WT mice with RD at 0, 4, and 12 weeks. (D–F) WT mice with HFD+L-NAME at 0, 4, and 12 weeks. (G–I) TRPC6 KO mice with RD at 0, 4, and 12 weeks. (J–L) TRPC6 KO mice with HFD+L-NAME at 0, 4, and 12 weeks.

Figure S4

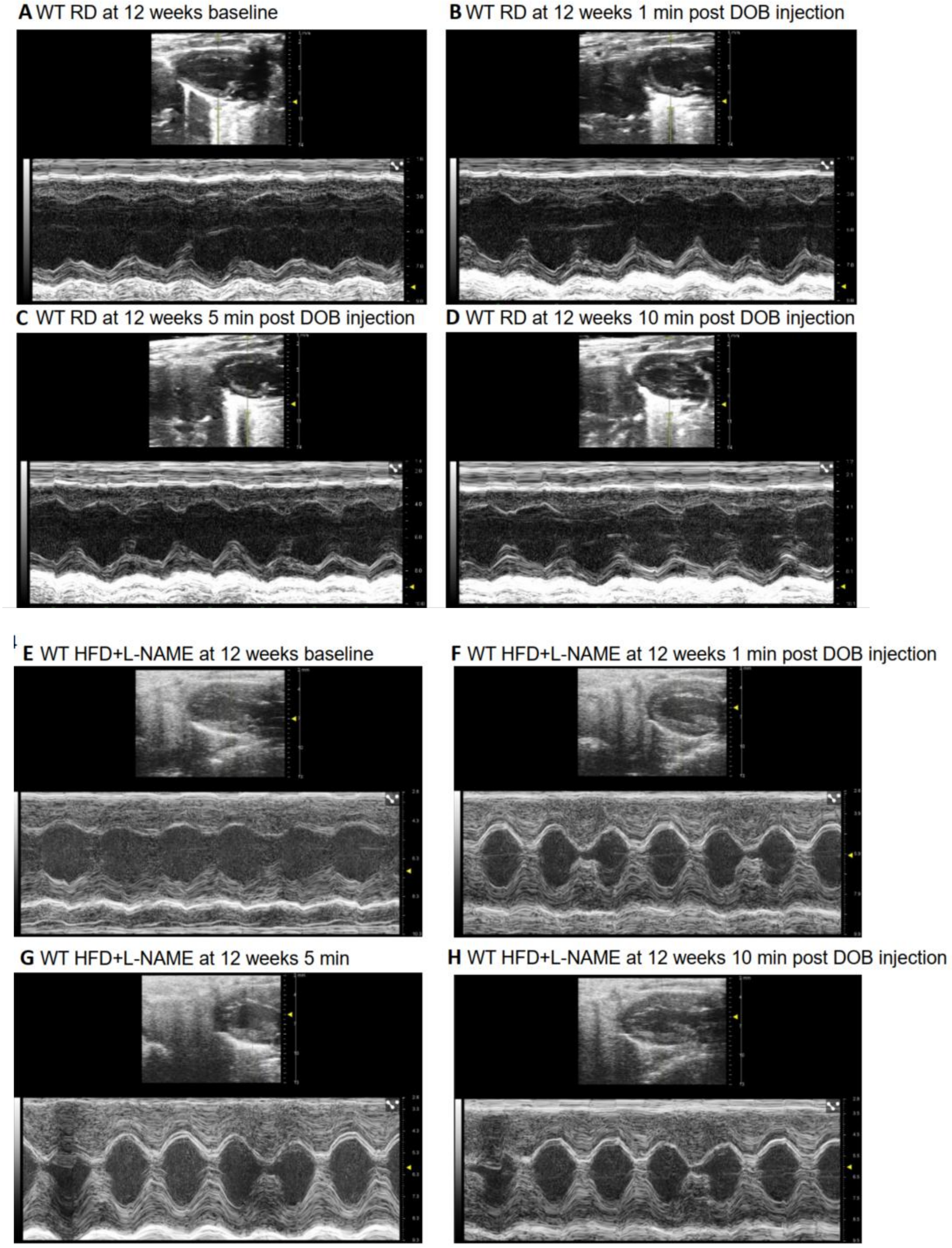
A WT RD at 12 weeks baseline
B WT RD at 12 weeks 1 min post DOB injection
C WT RD at 12 weeks 5 min post DOB injection
D WT RD at 12 weeks 10 min post DOB injection
E WT HFD+L-NAME at 12 weeks baseline
F WT HFD+L-NAME at 12 weeks 1 min post DOB injection
G WT HFD+L-NAME at 12 weeks 5 min
H WT HFD+L-NAME at 12 weeks 10 min post DOB injection

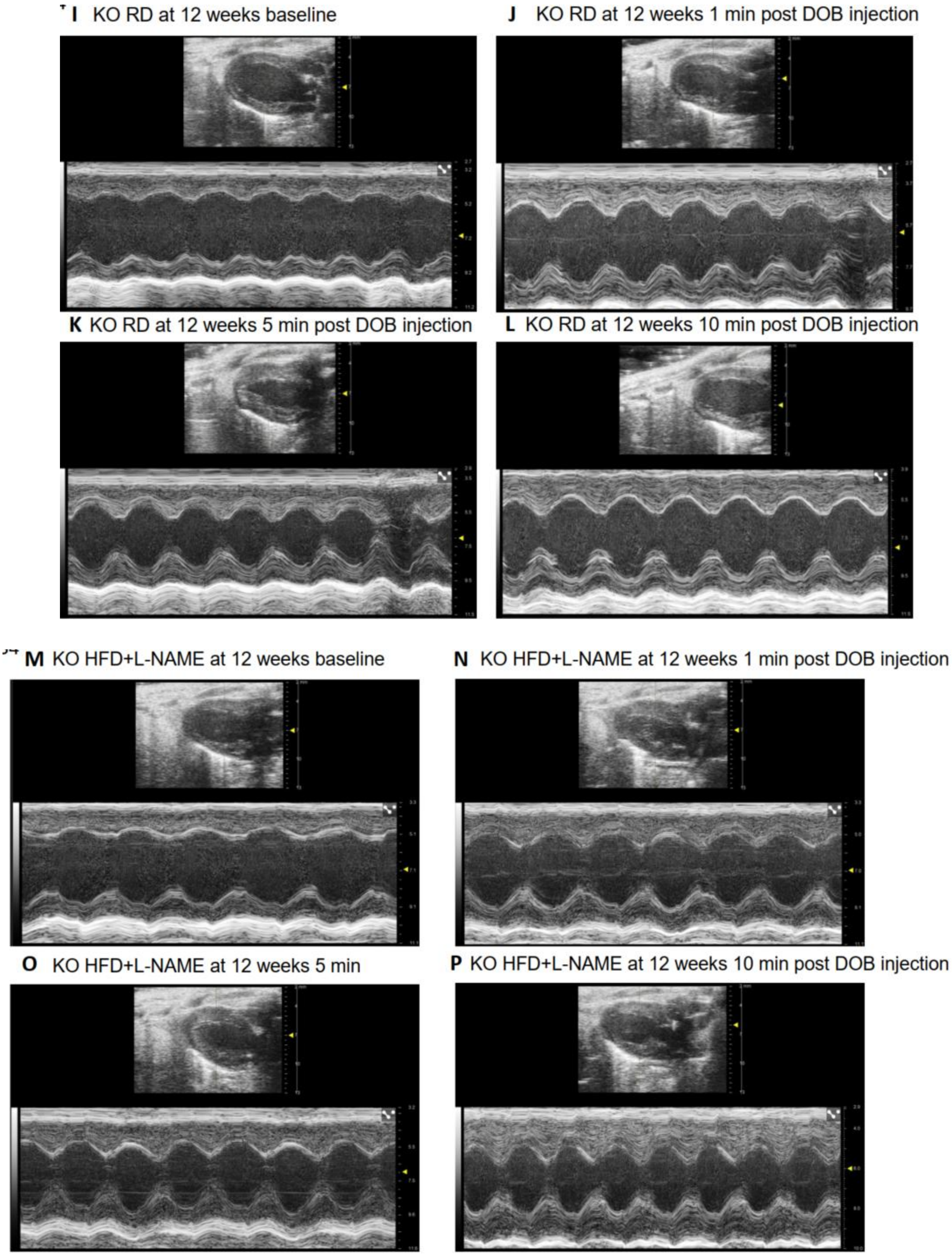

Figure S4. Representative echocardiographic B-mode (top) and M-mode (bottom) images of WT and TRPC6 KO mice before and after dobutamine treatment. (A–D) WT RD mice at baseline, 1, 5, and 10 minutes after injection. (E-H) WT HFD+L-NAME mice at baseline, 1, 5, and 10 minutes after injection. (I-L) TRPC6 KO RD mice at baseline, 1, 5, and 10 minutes after injection. (M-P) TRPC6 KO HFD+L-NAME mice at baseline, 1, 5, and 10 minutes after injection.
