# Supplementary material for: TRPC6 Deficiency Attenuates Mitochondrial and Cardiac Dysfunction in Heart Failure with Preserved Ejection Fraction Induced by High-Fat Diet Plus L-NAME
Source: Int J Mol Sci. 2025 Sep 25;26(19):9383. doi: 10.3390/ijms26199383 (PMC12524895; doi:10.3390/ijms26199383)
Supplement: Supplementary file 1 [file ijms-26-09383-s001.zip › ijms-3624736-supplementary.pptx]

## Slide 1
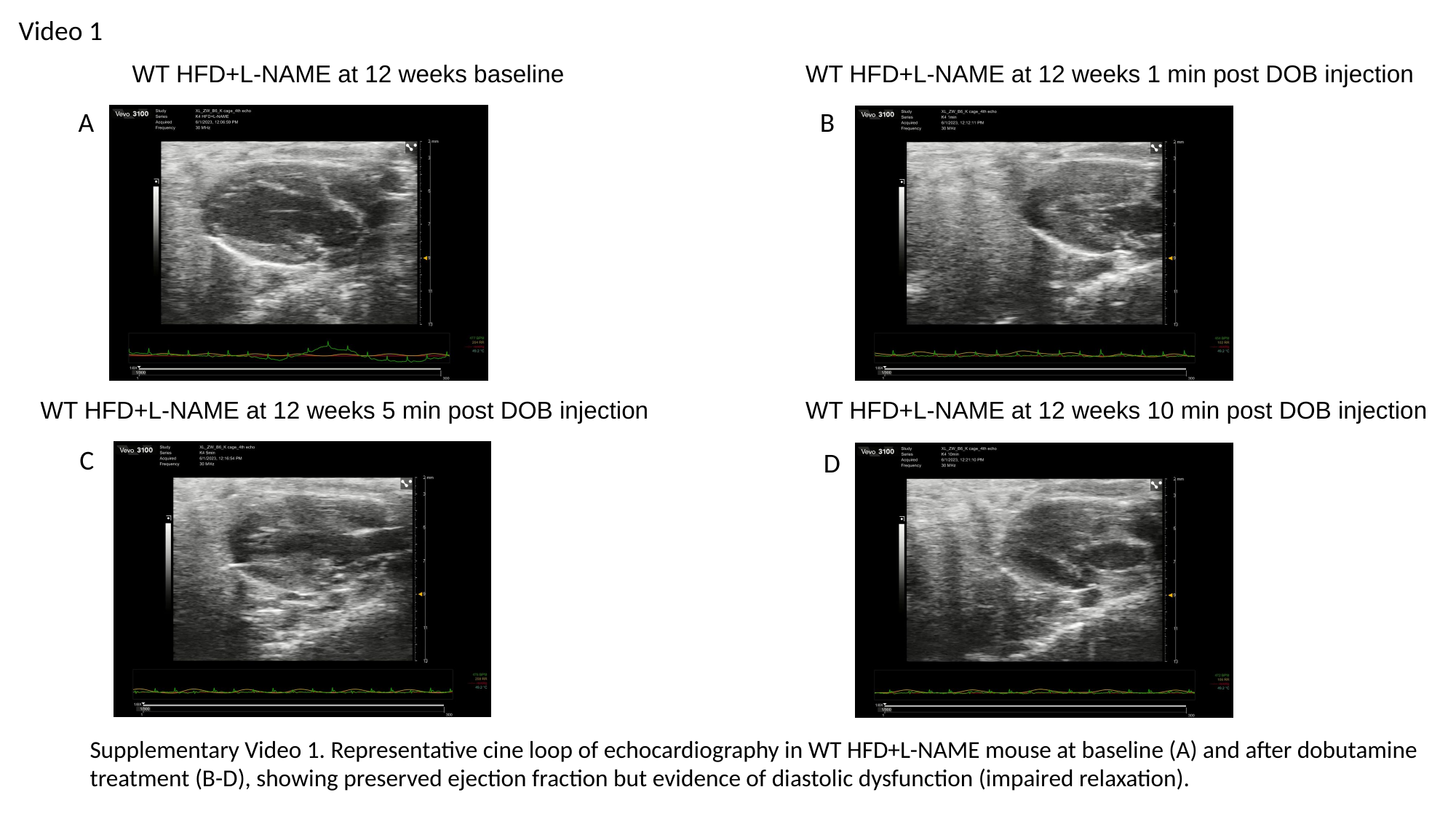

Video 1
WT HFD+L-NAME at 12 weeks baseline
WT HFD+L-NAME at 12 weeks 1 min post DOB injection
A
B
WT HFD+L-NAME at 12 weeks 5 min post DOB injection
WT HFD+L-NAME at 12 weeks 10 min post DOB injection
C
D
Supplementary Video 1. Representative cine loop of echocardiography in WT HFD+L-NAME mouse at baseline (A) and after dobutamine treatment (B-D), showing preserved ejection fraction but evidence of diastolic dysfunction (impaired relaxation).

## Slide 2
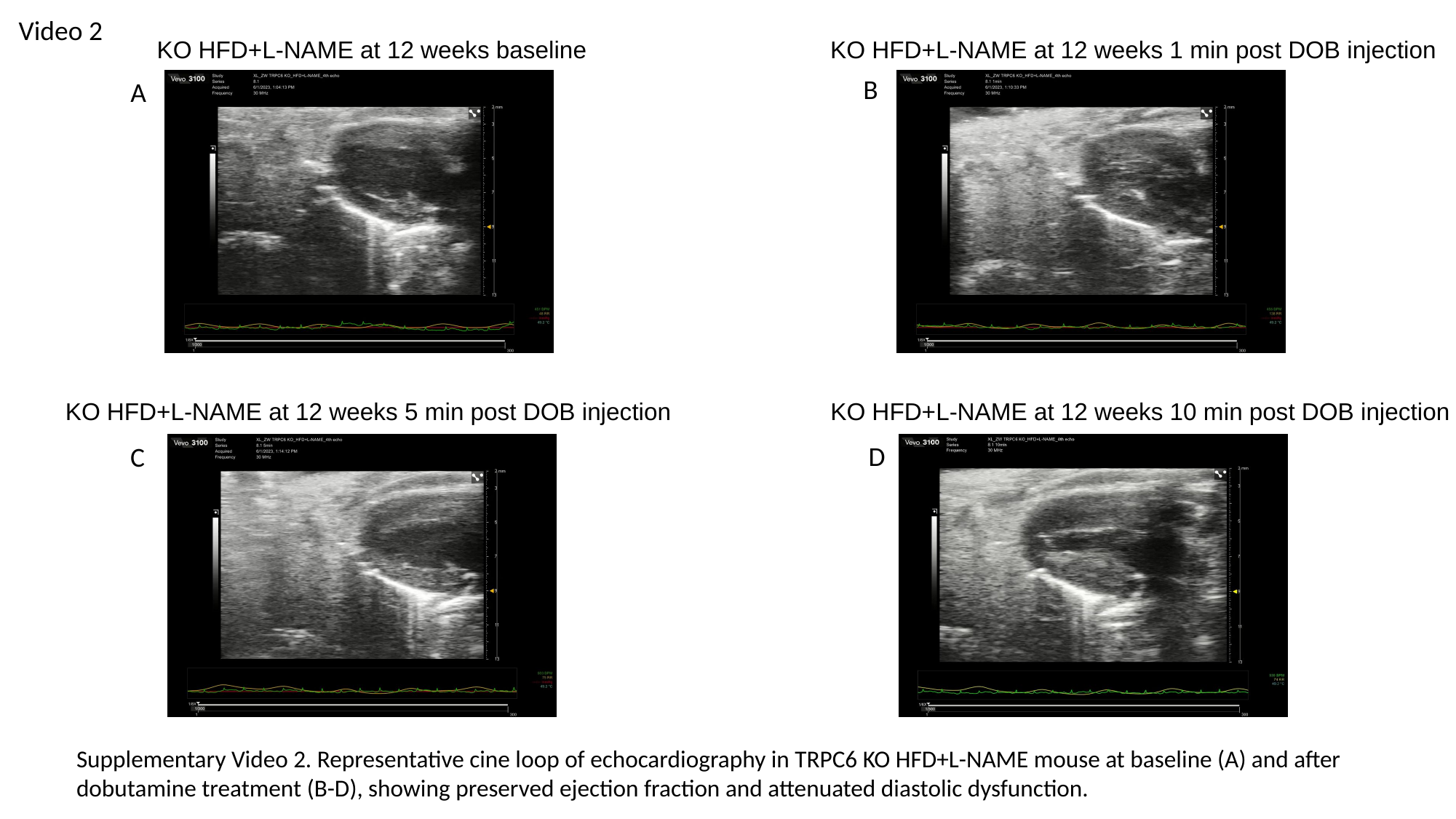

Video 2
KO HFD+L-NAME at 12 weeks baseline
KO HFD+L-NAME at 12 weeks 1 min post DOB injection
B
A
KO HFD+L-NAME at 12 weeks 5 min post DOB injection
KO HFD+L-NAME at 12 weeks 10 min post DOB injection
D
C
Supplementary Video 2. Representative cine loop of echocardiography in TRPC6 KO HFD+L-NAME mouse at baseline (A) and after dobutamine treatment (B-D), showing preserved ejection fraction and attenuated diastolic dysfunction.
